# A fast fabrication of copper nanowire transparent conductive electrodes by using pulsed laser irradiation

**DOI:** 10.1038/s41598-017-15559-3

**Published:** 2017-11-08

**Authors:** Nguyen-Hung Tran, Thanh-Hung Duong, Hyun-Chul Kim

**Affiliations:** High Safety Vehicle Core Technology Research Center, Department of Mechanical and Automotive Engineering, Inje University, Gimhae-si, South Korea

## Abstract

Copper nanowires have shown promise for use in next-generation conducting materials for transparent electrodes owing to their low sheet resistance, natural abundance, and high transmittance properties. Additionally, copper nanowires can be easily synthesized via low-cost solution-based processes. However, copper requires a uniform film to coat the nanowires on the substrate and removing film former residue in the post-treatment process remains a challenge. This lead to the high cost and complexity of fabricating transparent electrode. In this study, we demonstrate a simple, time-saving production method using a combination of laser irradiation and acid dipping to fabricate high-quality copper nanowire transparent electrodes. Preparation of electrodes was achieved by scanning pulsed laser on a copper nanowire film and then dipping in glacial acetic acid. The electrode exhibited excellent properties and the film former was totally erased from the electrode surface. Moreover, to demonstrate their capability, the as-fabricated electrodes were applied in touch-sensor fabrication.

## Introduction

In recent years, the demand for touchscreens has increased rapidly, from large flat panels for televisions and laptops to small devices such as smartphones, smartwatches, and navigation devices. Currently in the touchscreen industry, indium tin oxide (ITO) films coated on glass hold the major market share for transparent conducting electrode (TCE) materials. However, ITO has some inherent drawbacks, such as the unstable supply of indium, the substantial material wastage in the coating process (only 30% ITO is deposited onto the substrate), and its brittle nature^1–6^. Hence, various types of transparent electrodes based on nanoscale materials have been developed to overcome these shortcomings^7–11^. Currently, copper nanowires (Cu NWs) have attracted the most attention for use in TCEs because Cu has high electrical conductivity, low cost, and stable global supply^12–15^. In addition, the synthesis and material deposition of the Cu NWs on the film are well-known solution processes that are not only time saving (up to 1000 times faster than ITO sputtering), but also cost effective. In 2005, Yu *et al*. reported that high-quality ultra-long copper nanowires can be synthesized at a large scale with a facile aqueous reduction route at low temperatures^16^. Later, Wiley *et al*. developed a nitrocellulose-based ink to optimize the Meyer rod coating of Cu NWs. Although the studies showed the remarkable performance of Cu NW thin films, toxic chemicals and expensive, complex post treatments were used as part of the synthesis process^17,18^. Recently, we reported the fabrication of Cu NW electrodes using Polyvinylpyrrolidone (PVP)-based ink as a simple, low cost, and green synthesis method. The Cu NWs were coated onto substrates and were placed in an oven at 105 °C for 30–60 min and then immersed in acetic acid for 17.5 min to remove any chemical residue and the film former in a simple and effective process^17,19^. In these studies, the post-treatment is time consuming and energy intensive. More importantly, the ink is not totally removed from the final products, and many nanowires remain embedded in the PVP-based ink. Therefore, the wire-wire connections are limited, and consequently, the sheet resistance of the Cu NW network is extremely high. In addition, in the fabrication of touchscreens or solar cells, the contact area between the Cu NW layer and the other coating layer is highly reduced. Hence, the efficiency of the electrode drops, making this method unsuitable for commercial manufacturing. Many attempts have been made to reduce the junction resistance using different fabrication techniques such as mechanical pressing^20,21^, thermal annealing^22^, and optothermal heating^23^.

Recently, it has been found that laser irradiation can be used to modify the nanowire structure by plasmonic welding at the junctions, resulting in increased electrical conductivity^12,23^. However, in these studies, the metal nanowires treated by these processes were distributed on the substrates without any adhesive agents. In the coating method, the film former must be used to facilitate an even distribution of Cu NWs on the substrate. The synthesized nanowires usually coalesce together with the film former, so it is difficult to fuse the wires owing to inhibition of the contact between the individual nanowires.

In this study, a novel combined post-treatment using laser irradiation and acid dipping was developed to decrease the fabrication time of Cu NW-containing transparent electrodes. First, the Cu NWs were dispersed in PVP-based ink and then coated on a substrate by a Meyer rod. Next, the coated Cu NW film was irradiated by a pulse laser to weld the nanowire junctions and to partially remove the film former. After that, to guarantee that the Cu NW network was free from unwanted chemicals, the film was dipped in acetic acid for 1 min. The prepared Cu NW-based electrodes displayed excellent performance. Furthermore, to demonstrate the performance and practical use of the as-fabricated electrodes, a simple capacitive touch screen panel was built using laser patterning.

## Results and Discussion

### Synthesis of the Cu NWs

Owing to its simplicity and speed, the Ethylenediamine (EDA)-mediated method developed by Duong *et al*. was used to synthesize the Cu NWs^17^. The length and diameter of the as-synthesized Cu NWs were measured via scanning electron microscope (SEM) images (see Supplementary Fig. S1). The average diameter and length of the as-synthesized Cu NWs were approximately 150 nm and 53 μm, respectively. Furthermore, XRD analysis showed that CuO and Cu_2_O were not formed during the synthesis, as shown in Supplementary Fig. S2.

### The Cu NW-based transparent electrode

**Figure 1:**
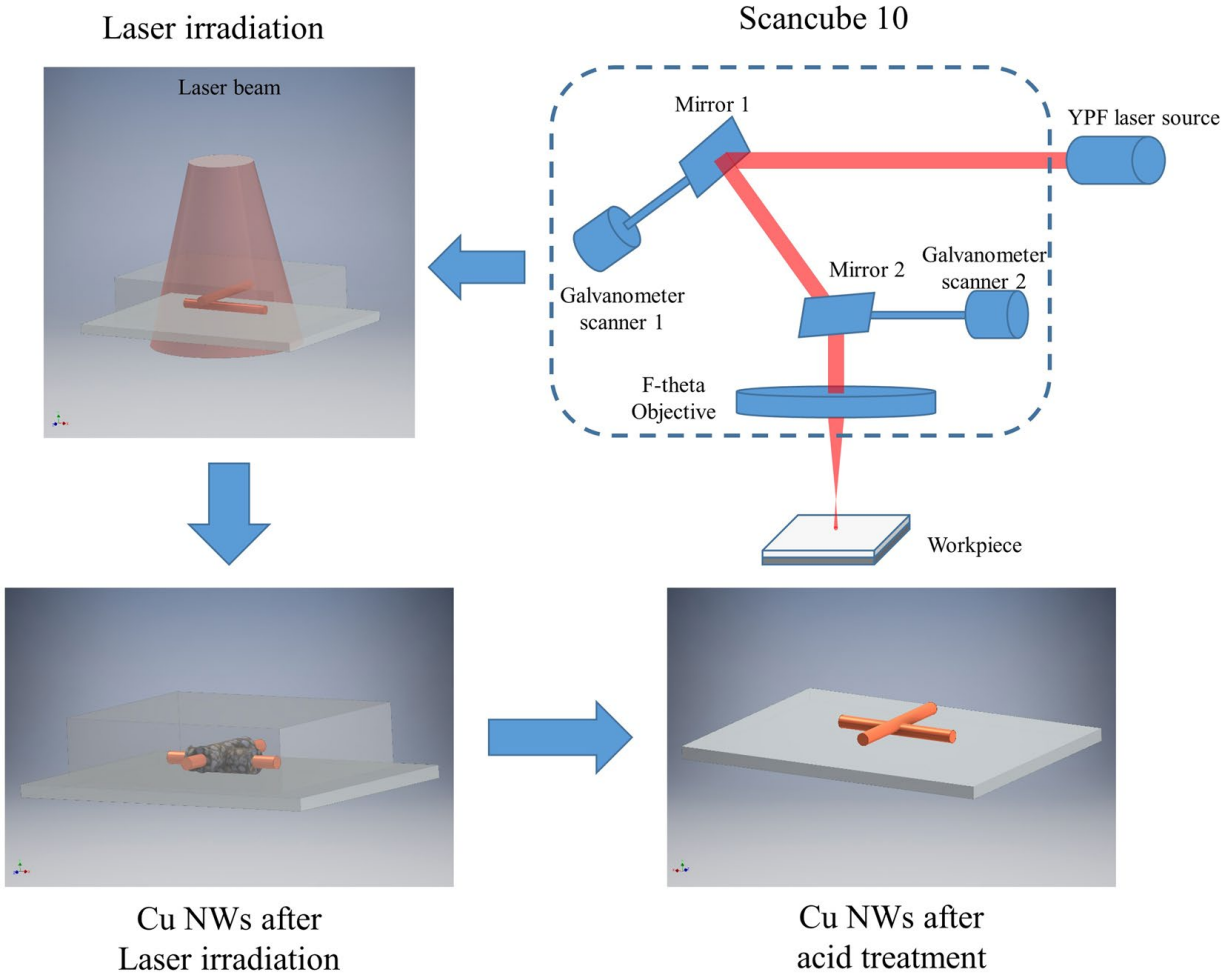
Fabrication principle of the Cu NWs transparent electrode using laser irradiation and acid treatment.

**Figure 2:**
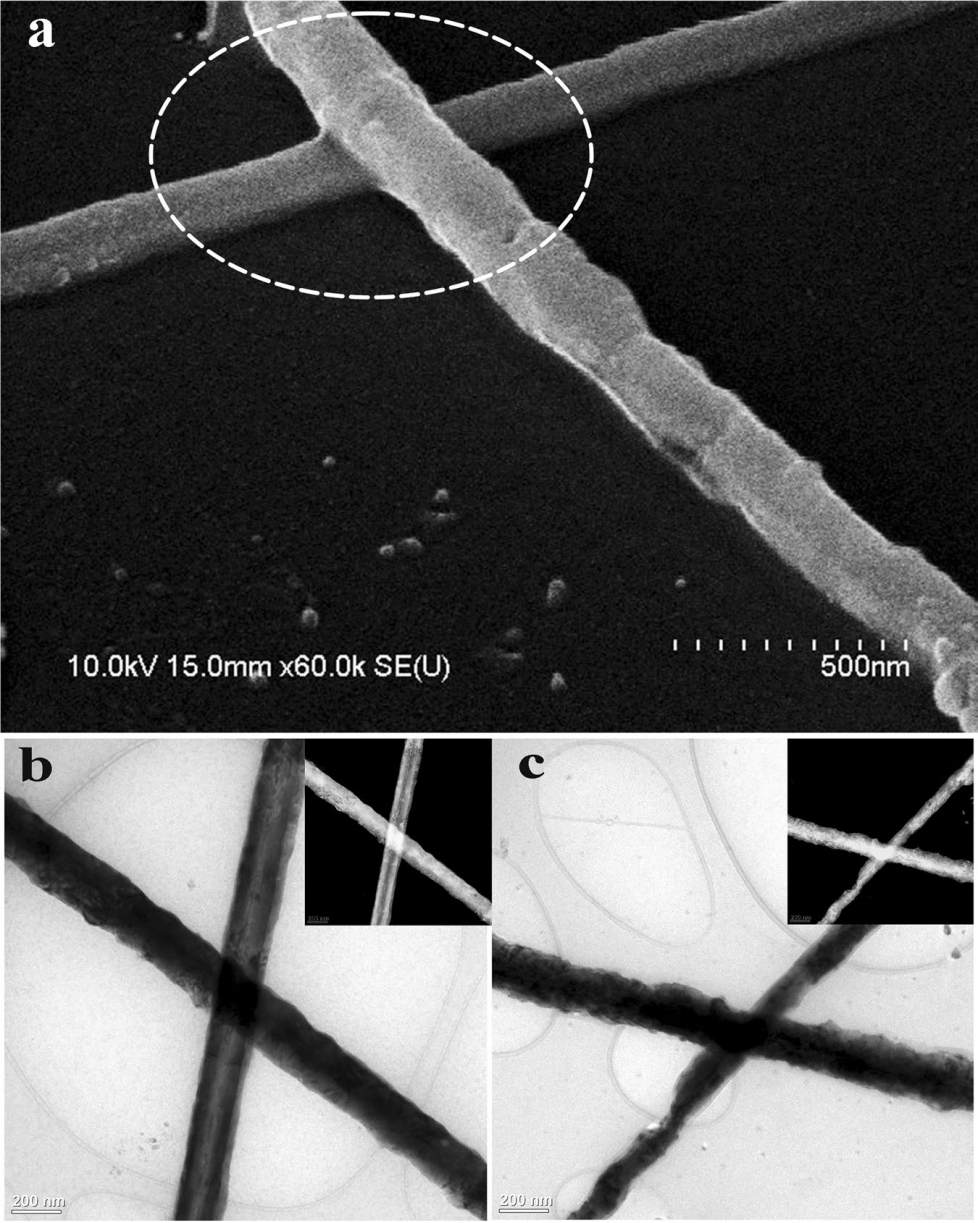
(**a**) The SEM image of the nanowelding by laser irradiation at the junction of two nanowires, the TEM images of (**b**) Cu NWs crossing before laser irradiation and (**c**) a fused Cu NWs junction after laser irradiation.

**Figure 3:**
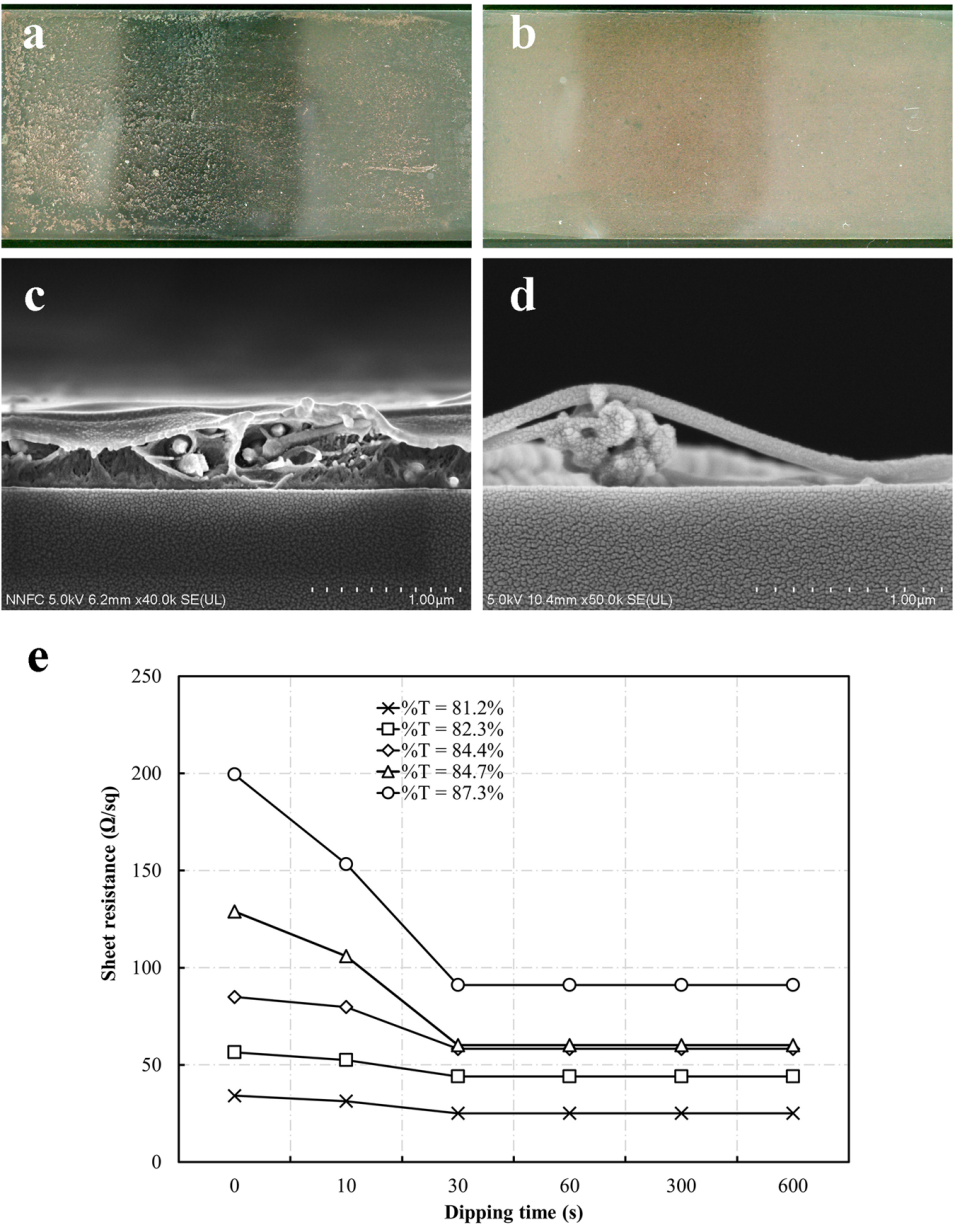
The effect of acetic acid treatment on the Copper nanowires. The transparent electrodes after (**a**) direct immersion in acid and (**b**) laser irradiation and subsequent immersion in acid. The cross-section of the (**c**) as-coated Cu NWs and (**d**) Cu NWs after acid dipping. (**e**) The sheet resistance of Cu NWs films with various dipping duration.

**Figure 4:**
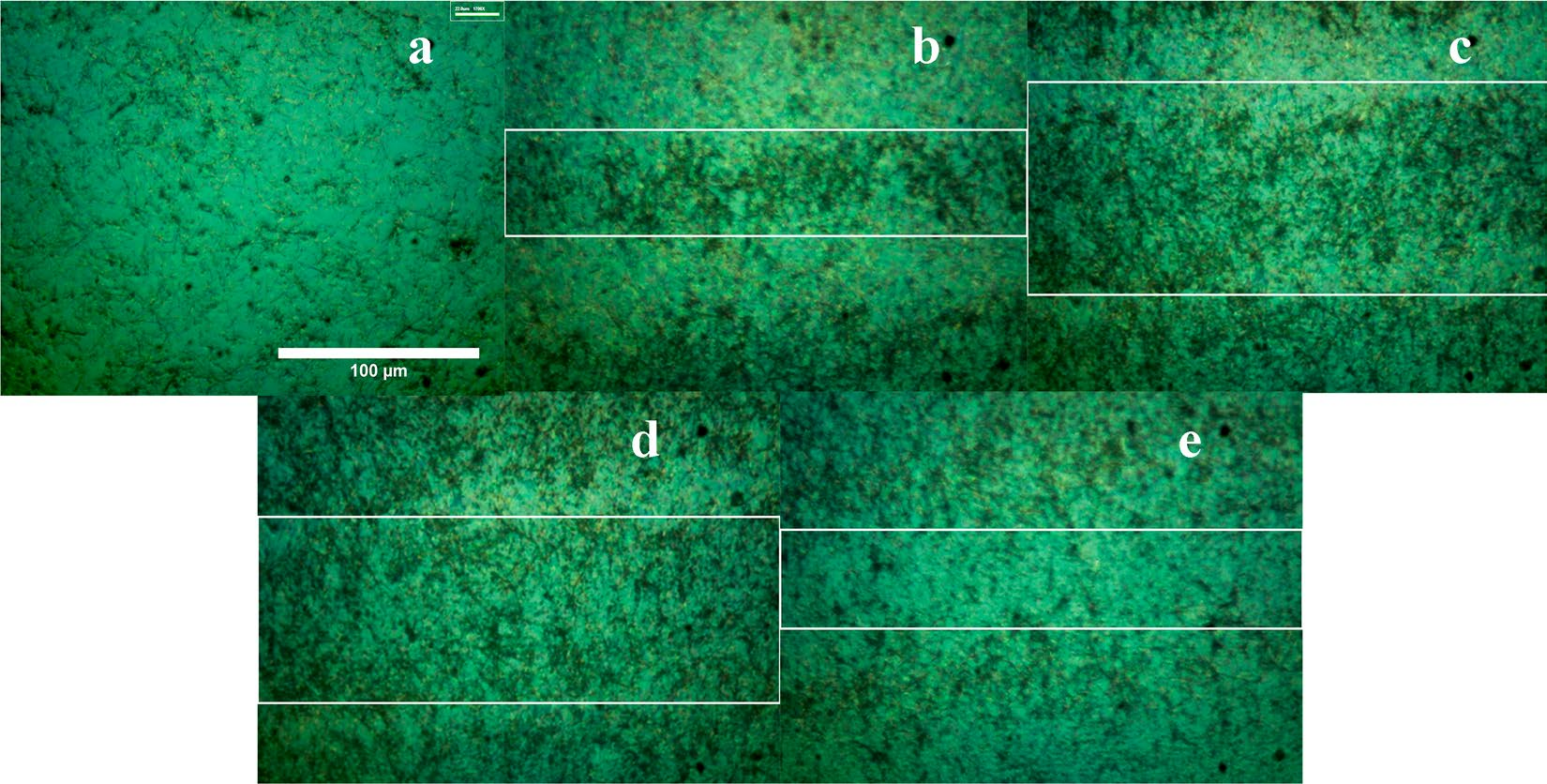
The effect of laser irradiation on the Cu NW percolation network. The optical images of copper nanowires irradiated with various laser power: (**a**) 0 µJ, (**b**) 4 µJ, (**c**) 8 µJ, (**d**) 12 µJ, (**e**) 20 µJ.

Cu NWs in a PVP-based ink were coated onto glass substrates (7.5 × 2.5 cm) using Meyer rod coating. As shown in Supplementary Fig. S1, the Cu NWs distributed evenly on the substrate surface. After coating on the glass substrates, the Cu NWs were covered by residual synthesis chemicals and PVP-based ink, resulting in a non-conductive film. If the acid treatment is used directly, the Cu NWs will be washed away immediately when removed from the acid solution. In the report by Willy *et al*., a solution to this problem was developed based on placing the coated Cu NW film on a hot plate and quick dipping in acetic acid. In a major advance in 2016, Duong *et al*. found that the Cu NWs moved downward and settled on the bottom of the PVP layer after heating in an oven for 1 h. Herein, we discovered that after irradiation by the pulse laser, the Cu NW electrodes could be dipped in glacial acetic acid for a long time without any network deformation, as illustrated in Fig. 1. The laser irradiation enhanced the nanowire contacts even for the Cu NWs buried in the PVP-based ink. Normally, the wire-wire contacts are established with local nanowelding because the plasmonic heating effect can be significantly augmented at the junction of metallic nanowires^12^. The fusing of the two nanowires at the intersection can be clearly seen in the SEM image shown in Fig. 2(a). Figure 2(b) and (c) compare the transmission electron microscopy (TEM) images of Cu NW before and after laser irradiation. Similar to the SEM image, the TEM analysis proves that the nanowelding was established at the cross of Cu NWs together. There are two main advantages of this laser irradiation process. First, because the wires were connected through nanowelding at the intersections, the conductivity of the electrode was improved remarkably. Second, nanowelding linked all the nanowires to create a solid network. Hence, the mechanical durability of the nanowire network was improved significantly, which prevented the loss of Cu NWs from washing with acetic acid (Fig. 3(a,b)). In addition, the thermal effect of laser irradiation can burn the film former covering the wires (Fig. 4(b,c)), which speeds up the removal of the film former by acid treatment.

To understand the behavior of the Cu NW network formed with the laser treatment, the as-synthesized Cu NWs were dispersed in a PVP-based ink to create a coating solution with a concentration of 20.9 mg/mL and were then coated using a Meyer rod bar. Next, the coated film was irradiated by a pulse laser. A range of pulse energies from 4 μJ to 20 μJ as well as the on/off (z = 3.5 mm) focal plane of the laser beam were investigated. After the coating process, the Cu NWs were embedded in the PVP-based ink, which is a transparent material, and the Cu NWs, which have a reddish color, can be seen in Figs 3(c) and 4(a). In case of focusing on the electrode surface, even with the minimum pulse energy, Cu NWs were ablated. The Cu NWs remained and turned to a darker color after being irradiated with defocusing at distance z = 3.5 mm from the focal plane and 4 µJ of pulse energy (Fig. 4(b)). Under the incident laser beam, only the nanowires were heated owing to the optical absorption properties of copper^24^. During rapid pulse laser heating on a nanosecond time scale, the Cu NWs absorbed the electromagnetic energy, and the nanowire temperature quickly increased, followed by heat diffusion from the wires to the surrounding ink^12,25^. As a result, the PVP-based ink around the Cu NWs melted and burned, and its color turned very dark. When the laser power was increased, the irradiated area expanded because of the lengthened Gaussian distribution of the laser beam. When the power of laser source rose above 12 μJ, the Cu NWs in the center of the laser beam were vaporized. This explains the brighter color of the center of the scanned line in Fig. 4(e).

**Figure 5:**
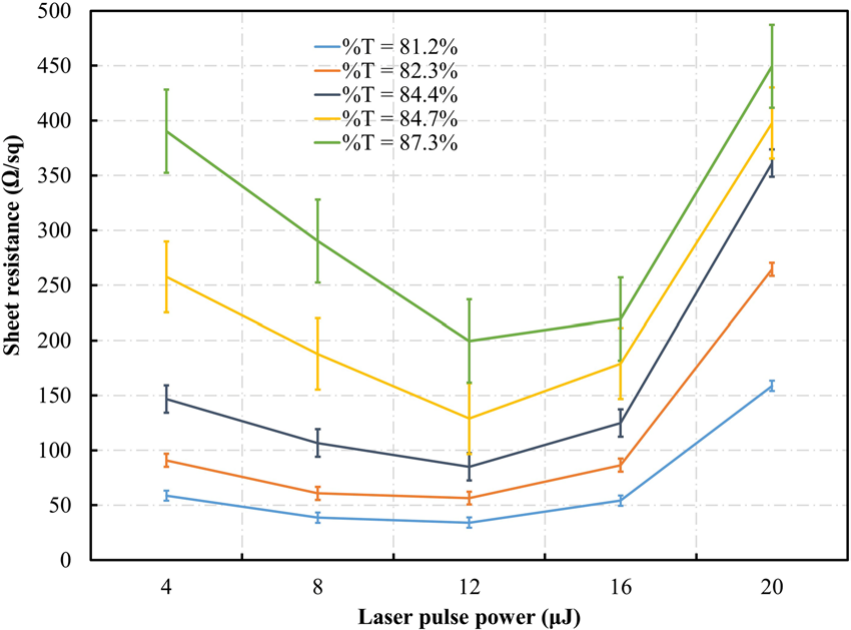
The sheet resistance of Cu NW network after laser irradiation with various laser power.

Next, to optimize the laser power in the irradiation process, five Cu NW coating solutions with different concentrations were prepared and each was coated with ink on five glass substrates using a Meyer rod. These samples were irradiated under a range of pulse powers from 4 to 20 µJ and then their sheet resistances and transparencies were measured. As shown in Fig. 5, after laser irradiation, all the Cu NWs coated on the glass substrates were conductive, proving that the wires were connected over the entire film. However, at 4 µJ, because the laser beam energy was too low to fuse the nanowire junctions completely, the conductivity of those electrodes was lower. When the pulse energy increased, the sheet resistance decreased significantly. All Cu NW films obtained the lowest sheet resistance at 12 μJ of laser pulse power. Above that energy level, the sheet resistance rose quickly, owing to the vaporization of the Cu NWs. The optical images shown that the coverage area of Cu NWs irradiated at 12 µJ of laser power is equivalent to the as coated Cu NWs. However, in case of the Cu NWs irradiated at 20 µJ of laser power (see Supplementary Fig. S3), it can be clearly seen that the nanowires concentration was decreased dramatically, which leads to reducing of the number of nanowire junction. As shown in Fig. 5, the sheet resistance declined from 58 Ω/sq to 34 Ω/sq for the sample with 81.2% transparency and from 390 Ω/sq to 199 Ω/sq for the sample with 87.3% transparency. Meanwhile, when the Cu NW films fabricated with higher Cu NW concentration was irradiated, there were more contact points, resulting in a lower sheet resistance and lower transparency. In addition, the transparency of TCEs was affected by laser scanning. As stated above, the burned film former causes a 10–15% decrease in electrode transmittance after the irradiation process. Based on the above results, to achieve the highest conductivity, the laser power was kept at 12 μJ during further experimentation.

Moreover, optimization of the dipping time was performed for the acid dipping process. A total of six Cu NW electrodes were prepared with different transparencies and irradiated by the laser beam for 4 ns, at a 120 kHz frequency, with 12 μJ of pulse power. The electrodes were immersed in glacial acetic acid for 10 to 600 s and then dried in air. It can be seen from Fig. 3(e) that the sheet resistance of electrode was reduced after dipping in glacial acetic acid. The lowest sheet resistances were obtained when the immersing time was longer than 1 min. Similarly, the transmittance values of the dipped films were significantly increased as the dipping time was raised up to a maximum at 60 s. Figure 3(c,d) show the SEM micrographs of the Cu NWs before and after acid dipping for 1 min. These images show that the PVP-based ink was entirely removed from the Cu NWs and substrates. Therefore, we determined that the optimum dipping time was 60 s. While the post-treatment technique of Stewart *et al*. takes 15 min and requires N_2_ gas and an oven^19^, and the Duong *et al*. method takes 76 min and requires an oven, our novel approach can treat a 5 mm × 2.5 mm electrode in 15 min under ambient conditions. More importantly, the film former was entirely removed.

**Figure 6:**
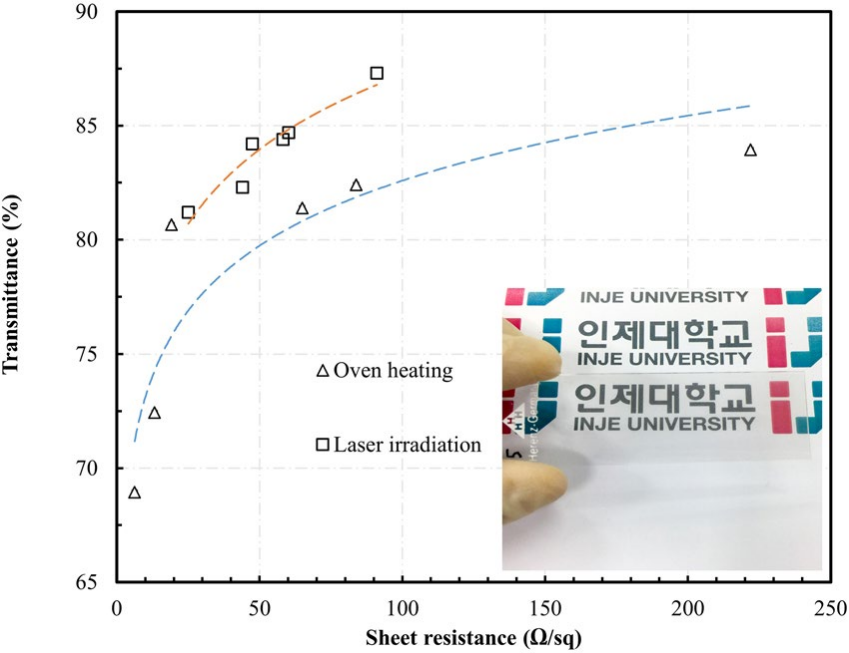
The plot of specular transmittance as a function of sheet resistance for the Cu NW films.

Next, to compare the performances of the oven heating and laser treatments, the Cu NWs synthesized with concentrations ranging from 12.5 to 18.7 mg/mL were used to fabricate the TCEs. The relation between sheet resistance and specular transmittance of the prepared electrodes is shown in Fig. 6. All the electrodes prepared by laser irradiation were superior to those fabricated by oven heating. For instance, at a specular transmittance of 85%, the laser irradiation electrode exhibited a sheet resistance of 60 Ω/sq while that of oven heating electrode was 70 Ω/sq. These results suggest that the combined laser illumination and acid treatment improves the quality of the prepared TCEs.

**Figure 7:**
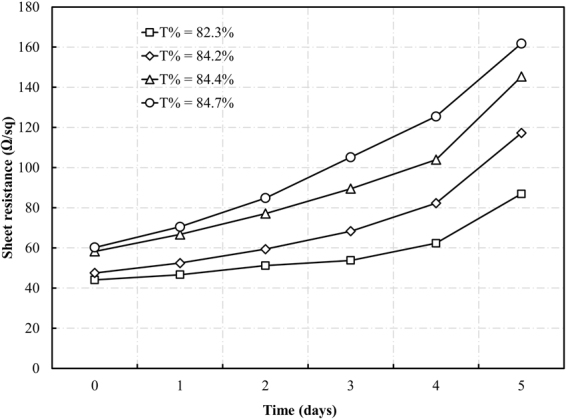
Stability of the Cu NW transparent electrodes with various transmittances.

Additionally, to determine the oxidation rate of fabricated Cu NW electrodes, four Cu NW transparent electrodes with different transparencies were fabricated using different concentrations of coating solution. The conductivity was examined daily, and after five days, the sheet resistances of all 4 samples increased to nearly 3 times the original value (Fig. 7). By the support of laser illumination process, the PVP-based ink was totally removed and only Cu NWs remained on the substrate surface after acid treatment. Hence, the nanowires were easily oxidized due to the direct contact to the oxygen in the air.

### Cu NW patterning and application in a touch sensor

**Figure 8:**
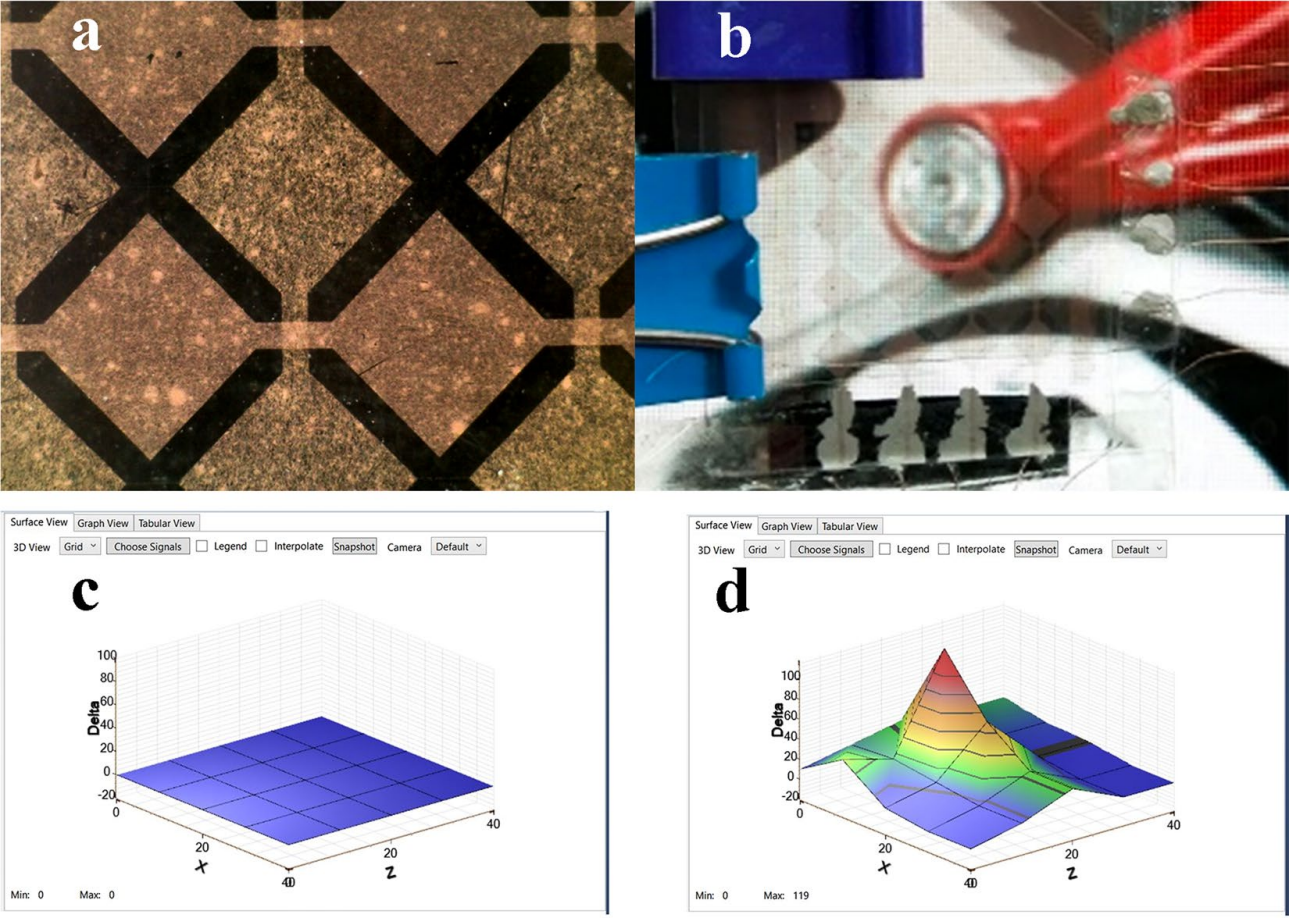
(**a**) The microscope of diamond-shaped patterning by laser ablation, (**b**) the fabricated touch sensor, (**c**) the sensor status without a finger touch, and (**d**) the sensor status with the approaching finger.

To exhibit the applicability of our TCEs, a touch sensor was made from the as-fabricated electrodes. First, two Cu NW transparent electrodes (25 Ω/sq of sheet resistance and 81.2% specular transmittance at 550 nm) were fabricated. Then the conductors were patterned with diamond shapes by maintaining the optimized laser parameters from the irradiation process and changing the focus point from defocused to on-focused. The total time required for the ablation was less than 1 min. After that, the electrodes were sandwiched with a 125 µm Polyethylene terephthalate film as a dielectric film and connected to a flash microcontroller (ATSAMD20J18, Atmel). As illustrated in Fig. 8, through the testing module, the sensor can detect the finger approaching the surface of the patterned conductors.

It can be concluded that a highly conductive and transparent copper nanowire film was produced via the combined post-treatment of laser irradiation and acid dipping. This method only took 15 min to treat the Cu NW transparent electrodes under ambient conditions and completely removed the film former on the substrate. By creating nanowelds at the wire-wire junctions, which was previously difficult to achieve owing to the embedding of the Cu NWs in the film former, the overall resistivity of the TCEs was reduced and the deformation of the network after dipping in acid was suppressed. Furthermore, the laser pulse energy as well as the dipping duration were optimized to obtain maximum performance. Finally, a touch sensor was fabricated using the prepared materials to confirm the potential of our approach in the touch screen industry.

## Methods

### Materials

Potassium hydroxide (KOH, 6597–4400), acetic acid (1002–4400), isopropyl alcohol (IPA, 5035–4400), and copper chloride (CuCl_2_) were purchased from Daejung Chemical & Metal (South Korea), Ethylenediamine (EDA, E1521), 35 wt.% hydrazine (N_2_H_4_) in water, polyvinylpyrrolidone (PVP, MW = 90000) were procured from Sigma-Aldrich (USA). Copper (II) chloride dihydrate (CuCl_2_.2H_2_O) was purchased from Junsei (Japan).

### Synthesis of copper nanowires

KOH (40 mL, 15 M), CuCl_2_ (2 mL, 0.1 M), and EDA (266 μL) were added to a reaction flask and heated to 60 °C and stirred at 700 rpm for 3 min. Next, N_2_H_4_ (35 wt%, 21 µL) was added to the mixture. After 2 min, the growth solution was stored at room temperature for 15 min without stirring. Later, a Cu NW disk formed and floated to the top of the solution. It was then transferred to a 50-mL tube for washing 3 times with 10 mL deionized water and centrifugation at 2000 rpm for 5 min.

### Preparation of the flexible transparent electrode

To prepare the coating ink, 2.5 g of PVP-K90 was dissolved in 97.5 g of IPA. The as-synthesized Cu NWs in 1 mL of IPA solution were then transferred to a 1.5 mL tube. Next, the nanowires were dispensed in IPA by vortex for 30 s to create a homogeneous solution, then centrifuged at 2000 rpm for 5 min to remove IPA. Lastly, depending on the desired concentration, the required amount of 2.5 wt.% PVP-k90 in IPA (from 0.6 to 1.2 mL) was pipetted into the copper nanowire tube to prepare the final coating solution (concentration from 12.5 to 18.7 mg/mL).

The prepared Cu NWs were mixed with a PVP-based ink and dispersed randomly on a glass substrate by Meyer rod coating (30.8 μm of wet thickness) to form a percolation network. After drying in air, the Cu NW network was scanned by a 1064 nm ytterbium pulsed fiber laser. The laser had an f-theta lens with f = 100 mm. The two electrically driven galvanometer mirrors inside the scan head (SCANcube® 10 ID# 116028) changed the laser direction, and adjusted the scanning speed and moving direction. The controllable Z-axis of a CNC stage enabled us to obtain an accurate focal point of the laser pulse focused or defocused on the nanowire network. Table 1 gives the scanning parameters used for the laser irradiation and ablation steps. The pulse frequency was controlled at 120 kHz to generate overlapped scanning. The laser pulse energy was varied from 4 μJ to 20 μJ to find the optimum value for the irradiation and ablation processes.

**Table 1:** Operation parameters for Cu NWs post-treatment.

Operation parameters	Value
Laser pulse energy	4 µJ, 8 µJ, 12 µJ, 16 µJ, and 20 µJ
Laser frequency	120 kHz
Laser pulse durations	4 ns
Dipping time	10 s, 30 s, 60 s, 300 s, and 600 s

Finally, to completely remove the residual PVP and other undesired chemicals, the Cu NW electrodes were dipped in glacial acetic acid for 1 min.

### Characterization

The synthesized Cu NWs were analyzed using a scanning electron microscope (SEM, Hitachi S-4800), transmission electron microscopy (TEM). X-ray diffraction (XRD) of the Cu NWs was measured in the range of 2θ = 20–80° by step scanning on the Rigaku D/MAX-2500 diffractometer (Rigaku Co., Japan). The T60 UV-visible spectrophotometer and the NI cDAQ – 9178 were used to measure the optical transmittance and the sheet resistance of the Cu NW electrode, respectively.

### Data Availability

The datasets generated during and/or analysed during the current study are available from the corresponding author on reasonable request.

## Electronic supplementary material

Supplementary Information
