# Supplementary material for: A fast fabrication of copper nanowire transparent conductive electrodes by using pulsed laser irradiation
Source: Sci Rep. 2017 Nov 8;7:15093. doi: 10.1038/s41598-017-15559-3 (PMC5678115; doi:10.1038/s41598-017-15559-3)
Supplement: Supplementary file 1 — Supplementary Information [file 41598_2017_15559_MOESM1_ESM.pdf]

Supplementary Information

A fast fabrication of copper nanowire transparent conductive electrodes by using pulsed laser irradiation

Nguyen-Hung Tran[1], Thanh-Hung Duong[1, *], Hyun-Chul Kim[1, *]

[1]High Safety Vehicle Core Technology Research Center, Department of Mechanical and Automotive Engineering, Inje University, Gimhae-si, South Korea

* Corresponding Author; E-mail: duongthanhhung@inje.ac.kr,

mechkhc@inje.ac.kr, Tel: +82-55-320-3988, Fax: +82-55-324-1723

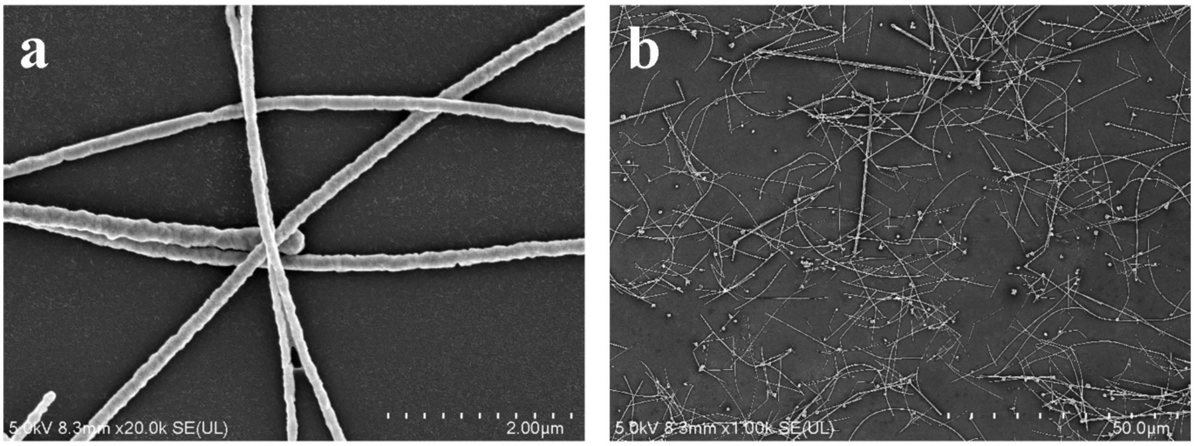

Fig. S1. SEM images of (a) synthesized Cu NWs and (b) the deposition of Cu NWs on a glass substrate the XRD of synthesized Cu NWs.

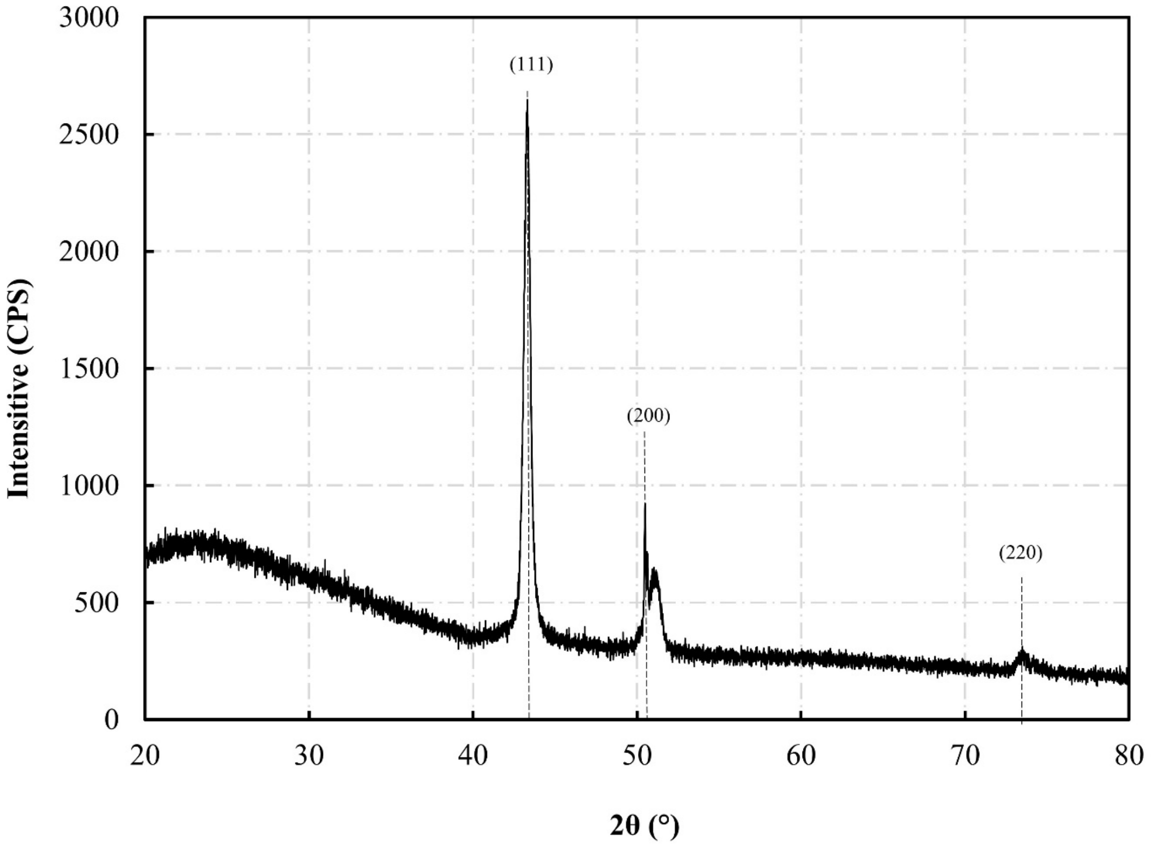

Fig. S2. The XRD of synthesized Cu NWs.

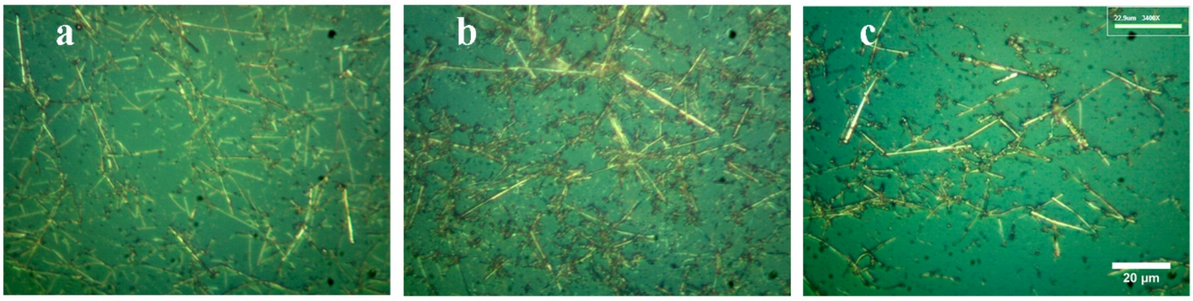

Fig. S3. (a) The as-coated Cu NWs on glass substrate. The Cu NW network after acid treatment with (b) 12 μJ and (c) 20 μJ irradiation laser power.
